# A Multi-Node Energy Prediction Approach Combined with Optimum Prediction Interval for RF Powered WSNs

**DOI:** 10.3390/s19245551

**Published:** 2019-12-16

**Authors:** Bikrant Koirala, Keshav Dahal, Paul Keir, Wenbing Chen

**Affiliations:** 1 School of Computing, Engineering and Physical Sciences, University of the West of Scotland, Paisley PA1 2BE, UK; keshav.dahal@uws.ac.uk (K.D.); Paul.Keir@uws.ac.uk (P.K.); 2 School of Mathematics and Statistics, Nanjing University of Information Science and Technology, Nanjing 210044, China; chenwb@nuist.edu.cn

**Keywords:** wireless sensor networks, energy harvesting, prediction, RF energy

## Abstract

Energy prediction plays a vital role in designing an efficient power management system for any environmentally powered Wireless Sensor Networks (WSNs). Most of the Moving Average (MA)-based energy prediction methods depend on past energy readings of the concerned node to predict its future energy availability. However, in case of RF powered WSNs the harvesting history of the main node along with neighbouring nodes can also be used to develop a more robust prediction technique. In this paper, we propose a Multi-Node energy prediction method for Radio Frequency Energy Harvesting (RF-EH) WSNs, which predicts the future energy availability by taking into account harvesting history of all nodes surrounding the main node. We analyse the effective distance for prediction and also develop a mathematical model to compute the optimum value of prediction interval, which has a major effect in prediction accuracy and system design, considering energy neutrality. Results show that Multi-Node prediction is less sensitive to prediction interval while inheriting the advantages of MA techniques. Also, nodes located at a larger distance were utilized less for prediction, and as the prediction interval increased, the utilization of more distant nodes decreased. Furthermore, we also establish a linear relation between the prediction interval and the energy threshold limit.

## 1. Introduction

In recent times, there has been a growing interest in the development of Wireless Sensor Networks (WSNs) that are capable of harnessing energy from ambient sources, such as solar, wind, vibration, heat and electromagnetic waves [1,2]. Radio Frequency Energy Harvesting (RF-EH) WSN has a sustainable power supply from ambient electromagnetic radiation, and thus opens up a new paradigm for powering energy-constrained WSNs [3]. RF-EH has the benefits of being wireless, available in the form of transmitted energy from various wireless devices, low cost, and small size [4]. Despite its advantages, the inherent challenge with this technique is the uncertain availability of ambient RF energy, which affects the energy efficiency of the WSN [5]. To mitigate this lag, it is crucial to know the energy harvesting profile in advance, so that the WSN’s energy utilization can be optimized based on future energy availability [6].

**Figure 1:**
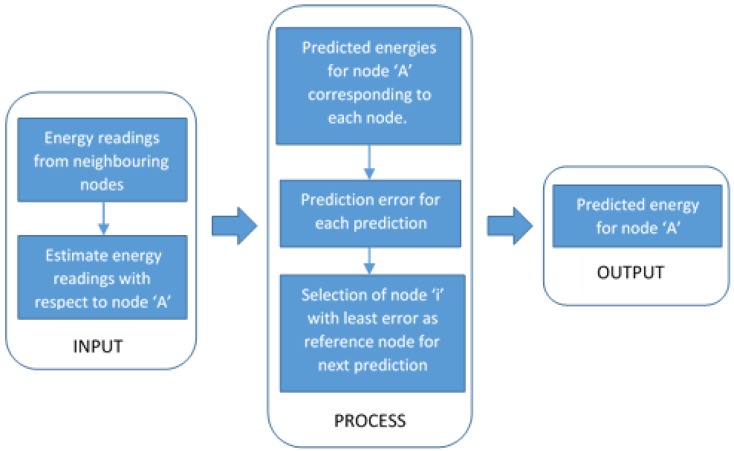
Multi-Node energy prediction model.

Many works focused on environmentally powered systems [7,8,9] have thus incorporated various energy prediction models to ensure best utilization of available energy by minimizing the likelihood of inactive periods due to lack of energy and energy wastage during high harvesting rate [10]. As RF energy suffers from phenomena such as reflection, diffraction, interference, scattering and others, its magnitude varies within small distances [11]. Such characteristics can be exploited in devising multi-node prediction models for RF-EH WSNs. Figure 1 depicts an energy prediction model based on multiple nodes, where energy prediction for a *Node-A* is performed not only by considering the past energy profiles of that particular node, but also by taking into account the harvesting history of its neighbouring nodes.

### Outline

In this paper, we present a novel energy prediction framework based on energy readings from multiple nodes for RF powered WSNs. In particular, we make the following contributions:

- We propose a Multi-Node energy prediction algorithm for RF-EH WSNs, which predicts future energy availability for a node by utilizing past harvested energy readings from surrounding nodes.
- We evaluate and compare the performance of our proposed prediction model with existing models for varying prediction intervals.
- We analyse the extent of the effective distance up to which a neighbouring node can be considered for prediction, and also the effect of prediction interval on the effective distance.
- We present a model for finding the optimum prediction interval ranges for RF-EH sensor nodes adopting a harvest-store-use mechanism for power management. The range is calculated considering Energy Neutral Operation (ENO) and the remaining energy in the sensor nodes.

The rest of the paper is organised as follows. In Section 2, we present a comprehensive review of related work. In Section 3, we propose a Multi-Node energy prediction model along with a description for obtaining the optimum range of prediction interval. Section 4 details the performance evaluation and comparisons accompanied by relevant discussions. Finally, Section 5 concludes the paper, followed by the References.

## 2. Related Work

In this section, we present the details of energy prediction approaches used for environmentally powered WSNs. Most of the energy forecasting methods are based on the Moving Average (MA) technique, which predicts the future value by taking an average of recent past values within a window of a certain length. In the Simple Moving Average (SMA) model, an average of the most recent *m* values is taken such that each past observation gets a weight of *1/m*. Hence, as m gets larger, it will yield a smoother-looking series of forecasts but will tend to lag behind in responding to trends and turning points. On the other hand, the Exponentially Weighted Moving Average (EWMA) model gives relatively more weight to recent observations than to older values. For this, a weighing factor, 0 < α < 1, is used as an exponentially decreasing weight on past values of a variable in order to perform a forecast [12,13]. 

A prediction algorithm based on weather conditions is described in [9] for solar-based energy harvesting. The proposed Weather-Conditioned Moving Average (WCMA) prediction algorithm is based on EWMA. WCMA differs from EWMA such that the former incorporates seasonal variations, which includes changes in the hour of sunrise and sunset and solar intensity differences. The evaluation for WCMA showed a reduction in prediction errors to as low as 10% compared to EWMA. Likewise, another prediction model, termed as Pro-Energy, developed for solar and wind energy harvesting WSNs was described in [10]. The model consists of three components—a prediction module, a profile analyser and a profile pool—and tries to match the current day’s observations with one of the profiles stored in its pool. The results demonstrated Pro-Energy prediction accuracies as high as 60% compared to EWMA and WCMA. Similarly, the work in [6] presented a comprehensive comparison of various solar energy prediction algorithms. From these comparisons, it was found that EWMA and the predictor developed at the Swiss Federal Institute of Technology of Zurich (ETHZ) occupied the least memory, while EWMA took the shortest time for prediction, with the average error in prediction being the smallest in the case of WCMA. These works, however, only present prediction models based on a single node, and do not consider harvesting history of the neighbouring nodes while forecasting future energy availability for a particular node.

There has been significant research in the area of prediction-based power management for EH networks. One such work is presented in [14], where the authors developed distributed methods to efficiently use harvested energy. The authors propose an energy-neutral and adaptive duty cycling algorithm based on EWMA energy prediction model. The result showed significantly higher performance levels, comparable even to the theoretical optimal calculated using complete future knowledge. The authors in [15] also proposed a network architecture for energy management in wind powered WSNs, based on future energy prediction. The architecture uses a weather forecast from the Internet for making predictions and deploys power management policies so as to ensure the autonomous operation of WSNs. The method used for prediction was similar to EWMA with adjustable weighing coefficients. The power management policy incorporates a dynamic period adaptation which is adjusted on the basis of state of the charge stored, predicted consumed and harvested energy. In [16], the authors presented a model based on the prediction of future available energy for optimizing problems related to buffer sizes, timing, and rates by adapting the parameters of a solar-powered WSN. The proposed model incorporates linear programming for capturing the performance, parameters, and energy model of energy harvesting systems. The literature described above does not provide sufficient insight for estimating an optimum prediction interval, which is crucial in developing prediction-based power management systems.

## 3. Basic Models and Methods

In this section, we present a Multi-Node energy predictor and modelling for optimum prediction intervals. In Section 3.1, we describe the proposed prediction method along with related theory and algorithm. Section 3.2 details our model for the optimum prediction interval range based on the RF-EH model described in [17]. 

### 3.1. Multi-Node Energy Prediction

**Figure 2:**
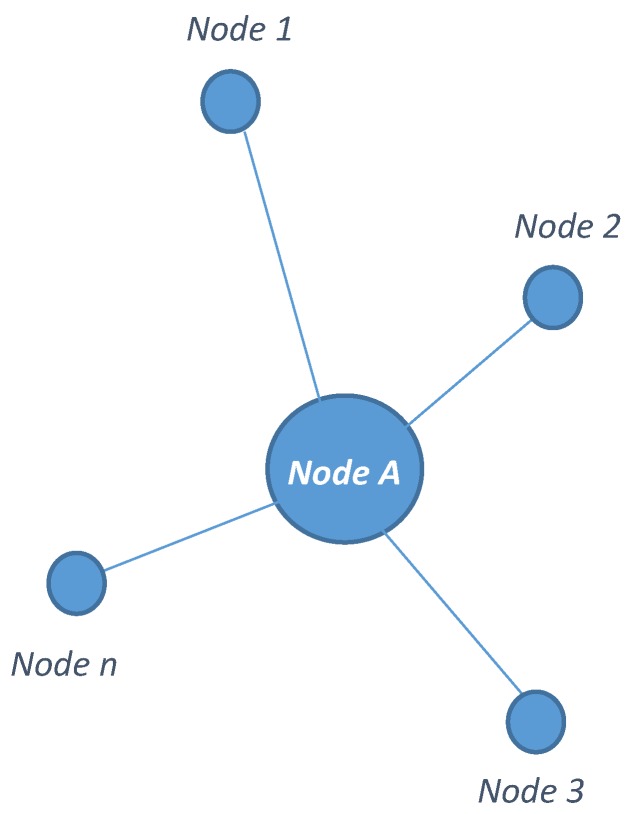
Node-A with neighbouring nodes.

The prediction model is based on the notion that there is significant variation in the magnitude of harvested RF energy even among the nodes lying within close vicinity. This phenomenon is due to the propagation characteristics of RF signals, and the degree of variation mainly depends on the frequency of the RF signal [11]. Figure 2 shows the nodes of an RF-EH sensor network, with *Node-A* surrounded by *n* number of nodes, namely *Node-1*, *Node-2*, *Node-3*, up to *Node-n*.

**Figure 3:**
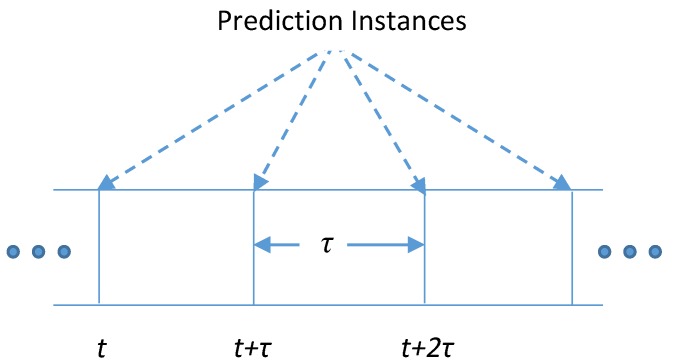
Time frame showing prediction instances.

The RF energy harvested by *Node-A* at any time instance can be predicted using the harvesting history of *Node-A* and its surrounding nodes. Figure 3 shows the prediction time frame with *τ* as the time duration between two consecutive predictions (prediction interval) and *t*, *t+τ*, *t+2τ* and so on as prediction instances. The theory behind the Multi-Node prediction method is that if the harvesting profile of neighbouring nodes for time *t* is known, it can be used to estimate the harvested energy for *Node-A* for that particular instance. The energy estimation for *Node-A* is carried out by assuming that all nodes hold location-based information, which is used to calculate the estimated energy using a suitable radio wave propagation model for any centre node (in this case *Node-A*) with respect to its neighbouring nodes. Using the estimated energy for *Node-A* corresponding to each neighbouring node, future energy availability is predicted for instance *t+τ***.** We will have *n+1* different versions of predictions for *Node-A* for all prediction instances. Next, the node with the least prediction error is chosen as the reference node for prediction instance *t+2τ* and so on.

In Step 4 of Algorithm 1, *e_iA_(t)*, *e_iA_(t- 1)*, *e_iA_(t-2)*,…, *e_iA_(t-m)* represent estimated energies for *Node-A* with respect to one of its neighbouring nodes *i* having the least prediction error. Prediction function *β*, which can be any of the MA-based prediction techniques, is then used to predict the future energy *ê_A_*(t+τ). For the next prediction cycle, prediction errors for *Node-A* are calculated as in Step 2 and the corresponding node giving the least error is selected for subsequent prediction and so on.
**Algorithm 1** Multi-Node energy prediction algorithmFor time *t*, predict energies for Node-A - *êA(t)*, *ê1A(t)*, *ê2A(t)*,…, *ênA(t)* with respect to *Node-A*, *Node-1*, *Node-2*, …, *Node-n*.Calculate prediction errors-*ɛA, ɛ1A, ɛ2A,…, ɛnA*. Where *ɛA* = Δ*{eA(t)-êA(t)}, ɛ1A = Δ{eA(t)-ê1A(t)}* and so on. Here, Δ is error function.Find the corresponding node *i* with least prediction error.Evaluate predicted energy for instance *t+τ*, i.e., *êA(t+τ)* = β*{ eiA(t), eiA(t- 1), eiA(t-2),…, eiA(t-m)}*. Here, *β* is prediction function.For time *t+τ*, repeat 2 and so on.

### 3.2. Optimum Energy Prediction Interval

The prediction accuracy depends on the prediction interval. As the prediction interval increases, the prediction error tends to decrease (discussed later in Section 4). Also, in the case of a harvest-store-use network architecture [3], it is crucial to make an energy availability prediction before the stored energy in the buffer is too low to power the necessary scheduled task. These situations call for the prediction interval to be bounded within a range so that predictions can be made as quickly as possible with an acceptable prediction error. For this, we utilize the RF-EH model presented in [17] to develop a mathematical formulation for estimating an optimum prediction interval range that can accommodate the above situations.

**Figure 4:**
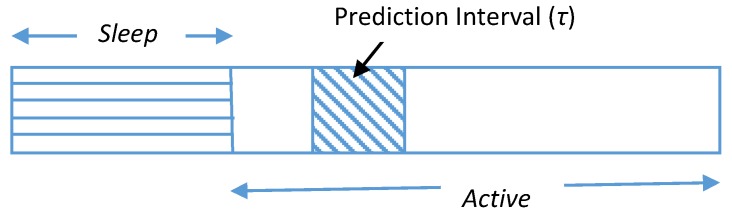
Prediction interval along with *Sleep* and *Active* states.

Figure 4 represents *Sleep* and *Active* states for a typical EH node [14]. During the *Sleep* state, an RE-EH node harvests energy from the source, stores it in the buffer and performs no tasks, i.e., during this state, a node ideally does not consume any energy. On the contrary, when the node is in the *Active* state, it performs the scheduled network operation alongside harvesting energy and storing it in the buffer.

If *e_sleep_* is the energy stored in the buffer during the *Sleep* state, then

*e_sleep_ = P_savg_ × t_s_* (1)

where *P_savg_* and *t_s_* represent the average RF energy harvesting rate and *Sleep* time respectively.

Likewise, for an arbitrary interval of Δ*t_a_* during the *Active* period, the energy stored in the buffer can be represented by *e_sb_* as,

*e_sb_ = e_i_ + P_savg_* × Δ*t_a_ − P_Lavg_* × Δ*t_a_* (2)

where *e_i_* is the energy stored in the buffer at the beginning of Δ*t_a_* and *P_Lavg_* is the average energy consumption rate when nodes are *Active*.

If we consider an arbitrary interval to always begin from the very start of the *Active* state, then *e_i_ = e_sleep_*. This assumption is made to avoid the chances that a node runs out of its energy before a prediction is made. Also, replacing *P_Lavg_* by the optimum energy consumption rate, *P_Lopt_* as described in [17] ensures an optimal utilization of the harvested energy maximizing the RF-EH WSN lifetime. We can then rewrite Equation (2) as follows

*e_sb_ = e_sleep_ + P_savg_* × Δ*t_a_ − P_Lop_* × Δ*t_a_* (3)

To maintain energy neutrality, the energy consumed in Δ*t_a_* should always be less than or, at worst, equal to the sum of stored energy and harvested energy during the interval. That is,

*P_Lopt_* × Δ*t_a_ ≤ P_savg_* × Δ*t_a_ + e_sb_* (4)

This leads to,

*P_savg_* × Δ*t_a_ + e_sb_ − P_Lopt_* × Δ*t_a_ ≥ 0* (5)

To be sure that the energy prediction is made well before the limiting condition given by Equation (5), we introduce a threshold energy level *k × e_sleep_* (where *k* is a threshold constant defined in the interval [0,1]), such that for a prediction interval of *τ* the following condition is satisfied,

*P_savg_. τ + e_sb_ − P_Lopt_ × τ = k × e_sleep_* (6)

Further, from Equation (6) we can derive,

(7) $$\frac{\tau }{t_{s}}=\;\frac{\left(k-1\right)P_{savg}}{2\left(P_{savg}-P_{Lopt}\right)}$$

Equation (7) provides a measure for the prediction interval *τ* based on an optimum energy consumption rate *P_Lopt_*, which ensures energy neutrality and minimal energy wastage. 

## 4. Results and Discussion

**Figure 5:**
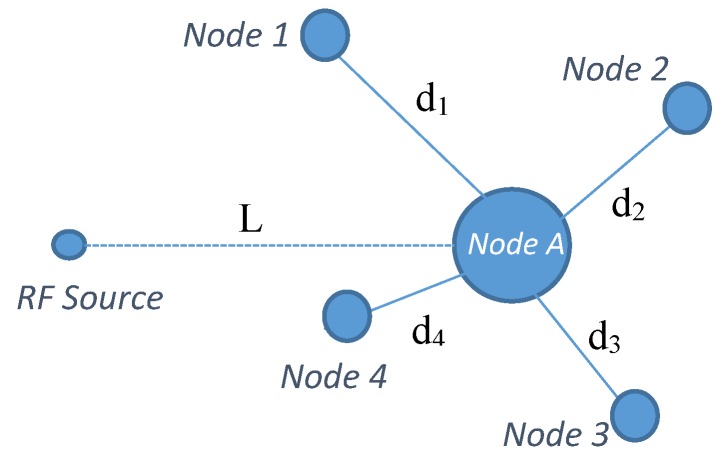
Simulation layout-1 for Multi-Node prediction.

The layout-1 of the simulated network considering four neighbouring nodes is shown in Figure 5. *Node-A* is the node of interest for which energy prediction is to be made with *Node-1, Node-2, Node-3* and *Node-4* as neighbouring nodes at a distance of d_1_, d_2_, d_3_ and d_4_, respectively. A single RF source at a distance L and with the specifications presented in Table 1 was considered for the simulation. For layout-1, the nodes were arranged such that the angle of separation between *Node-1/Node-2, Node-2/Node-3, Node-3/Node-4* and *Node-4/Node-1* were 60°, 40°, 20° and 240°, respectively, with *Node-A* being the centre node. Likewise, for layout-2, a star topology was formed with *Node-A* surrounded by eight nodes placed at 0.5 m, 2 m, 3.5 m, 4 m, 6 m, 8 m, 10 m and 12 m; with a 45° angle of separation between adjacent nodes.

The simulation was carried out in the MATLAB (R2017b) platform. RF source with omni-directional antenna and sinusoidal impulse response was linked with the individual sensor nodes through an AWGN (Additive White Gaussian Noise) channel to mimic the effect of random process. The choice of AWGN channel model was made, as it is best suited for static (non-mobile) energy harvesting sensor nodes [18]. A mean of 0 (*µ* = 0) and variance of 0.1 (*σ* = 0.1) were assumed for channel modelling. A free space path loss model was incorporated throughout the simulation to estimate the energy loss during the signal propagation. An ideal lossless buffer was considered, and the overall harvester efficiency was set to be 0.7, which included the efficiencies of antenna, rectifier and converter circuits combined. 

Our Multi-Node energy prediction algorithm was used to estimate the future energy availability for *Node-A*. For this, as discussed in Section 3.2, the estimated harvested energy for *Node-A* with respect to all the neighbouring nodes was used to compute the predicted energy values corresponding to each node (including *Node-A*). In our simulation, the Multi-Node energy prediction algorithm was implemented using SMA (*m* = 10) as the prediction function. The simulation was performed for varying prediction intervals. Table 2 shows the measurements of actual harvested energy along with predicted values for prediction interval of 60 s.

**Figure 6:**
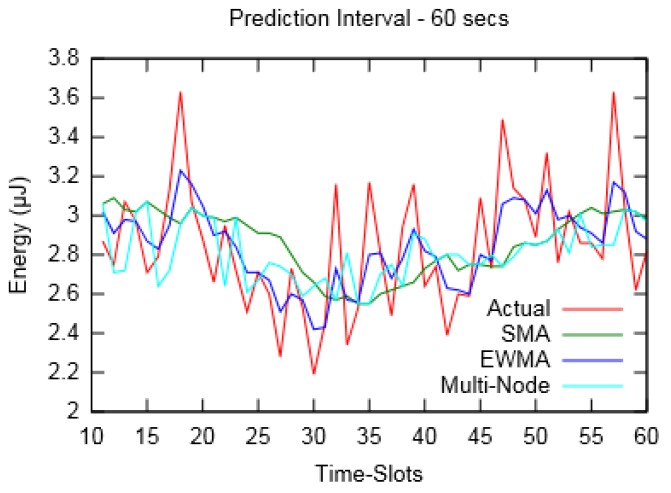
Actual and predicted energy for prediction interval of 60 s (for layout-1).

**Figure 7:**
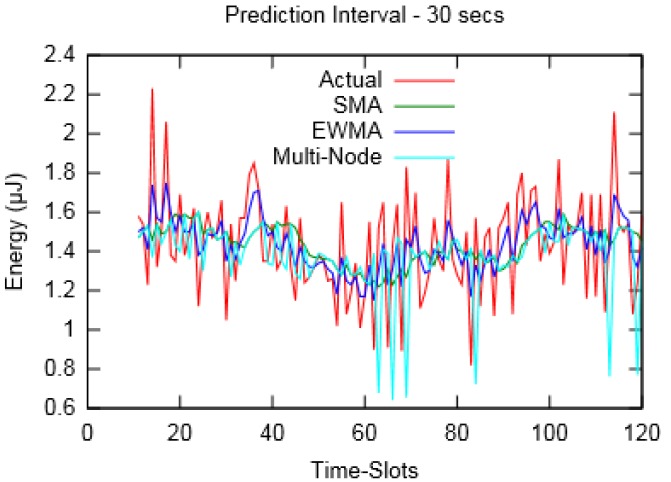
Actual and predicted energy for prediction interval of 30 s (for layout-1).

**Figure 8:**
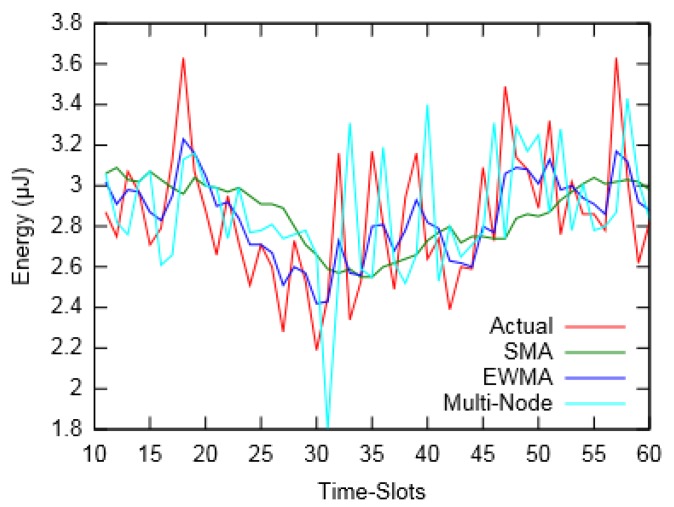
Actual and predicted energy for prediction interval of 60 s (for layout-2).

**Figure 9:**
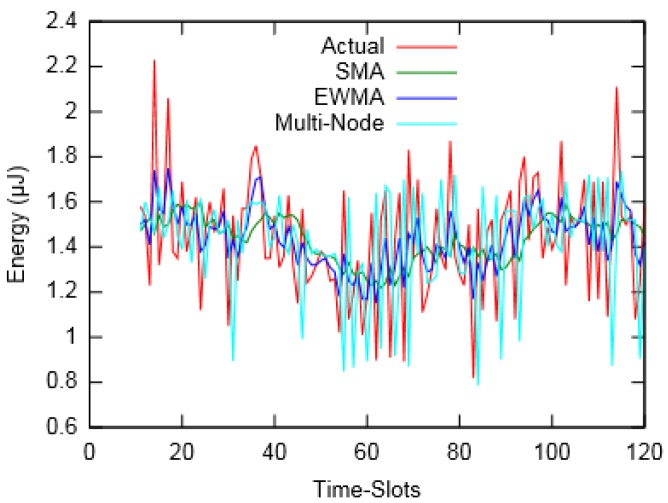
Actual and predicted energy for prediction interval of 30 s (for layout-2).

Figure 6 and Figure 7 depict the actual harvested energy by *Node-A* along with predicted energy using SMA (*m* = 10), EWMA (*α* = 0.4) and the proposed Multi-Node prediction algorithm. From the figures, it can be observed that the EWMA prediction approach follows the actual energy profile better than SMA and Multi-Node. However, in terms of Mean Absolute Percentage Error (MAPE), taken for a duration of an hour, SMA performed better compared to the other two. Similarly, Figure 8 and Figure 9 show the actual and predicted energy for *Node-A* considering eight neighbouring nodes. It is evident from Table 3 that as the prediction interval decreases, the Multi-Node prediction approach gives better results than other two approaches.

**Figure 10:**
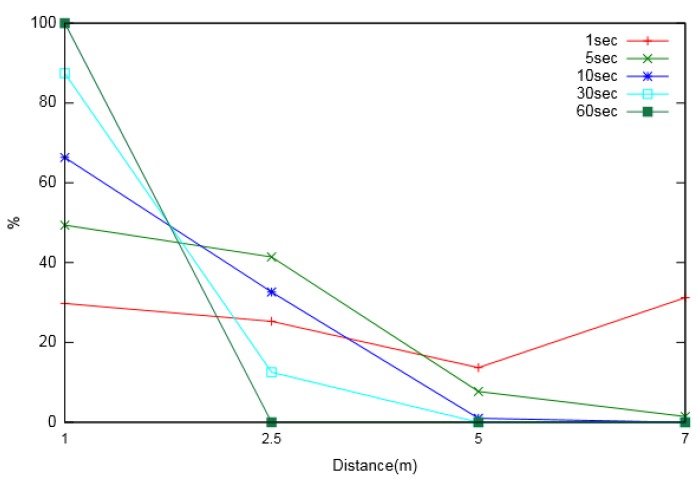
Neighbouring node usage percentage for different prediction intervals (for layout-1).

**Figure 11:**
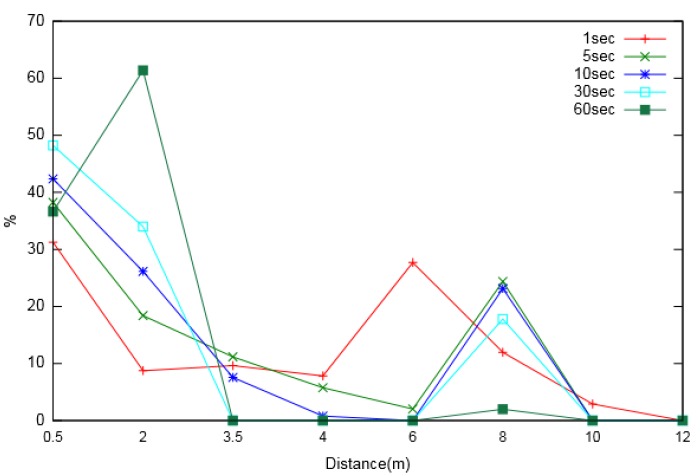
Neighbouring node usage percentage for different prediction intervals (for layout-2).

We also made observations for node usage during Multi-Node energy prediction. The analysis was carried out in order to understand the extent of the effective distance up to which a neighbouring node can be considered for prediction. The other reason for the observation was to understand the effect of the prediction interval on the effective distance. Figure 10 and Figure 11 show the percentage usage of neighbouring nodes located at various distances from the node of interest, for varied prediction intervals. Two major phenomena were observed from the analysis. First, nodes located at a larger distance were utilized less for prediction compared to nearer neighbouring nodes. Second, as the prediction interval increased, the utilization of more distant nodes decreased, suggesting that the extent of the considered neighbouring nodes was larger for smaller prediction intervals.

**Figure 12:**
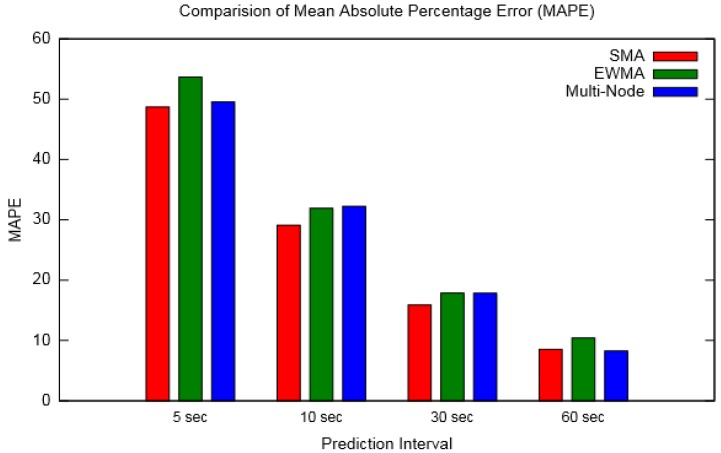
MAPE for various prediction intervals (for layout-1).

From Figure 12 and Table 3, it can be observed that prediction error decreased considerably for larger forecast durations. This implies that the prediction interval should be chosen so that the error is within acceptable limits, and the prediction should be made before the node energy falls below a threshold value, as discussed in Section 3.2. To find this optimum range of prediction interval, we first calculated the optimum energy consumption rate P_Lopt_ using the method detailed in [17], which was finally used in Equation (7).

**Figure 13:**
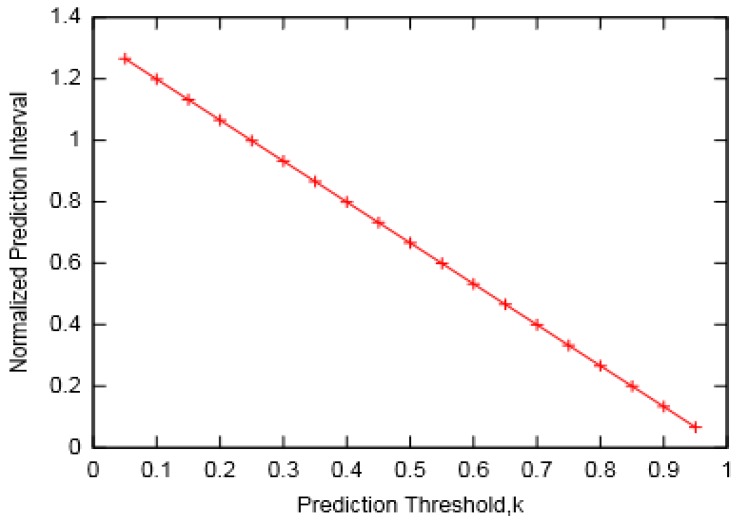
Prediction intervals for different threshold values.

**Figure 14:**
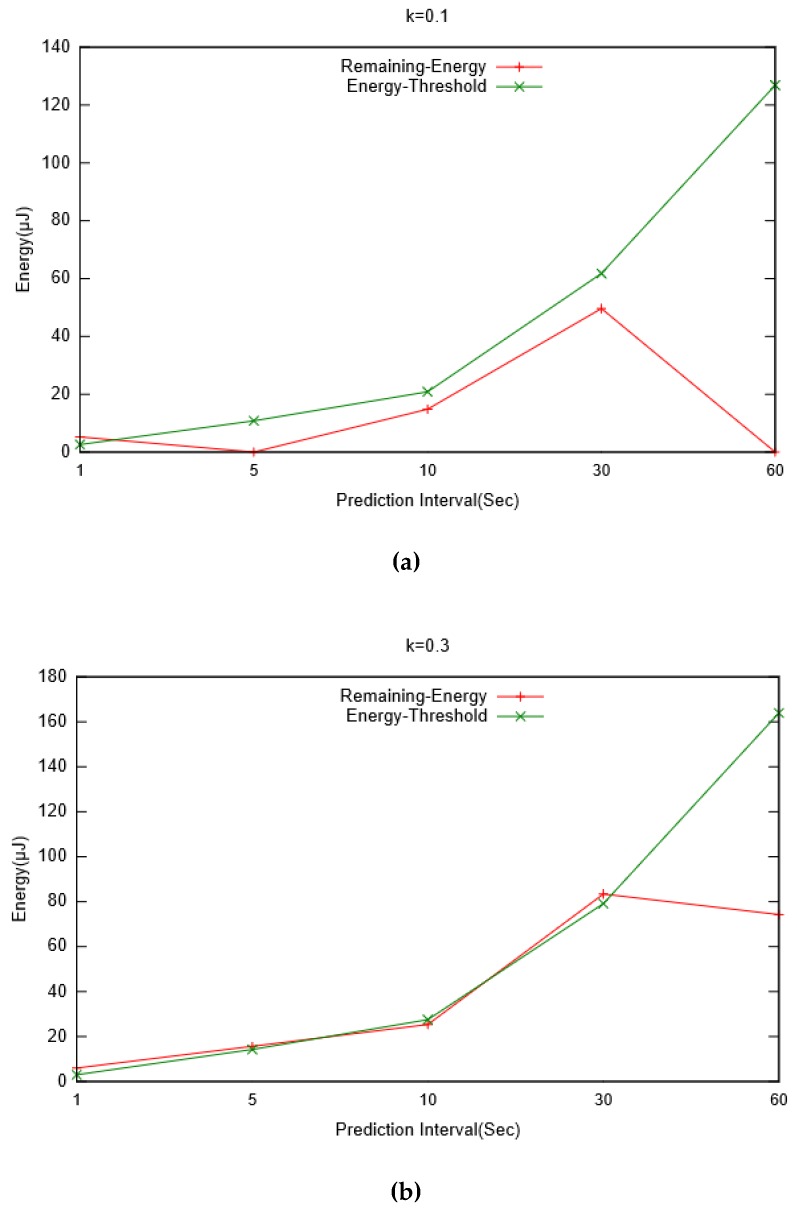

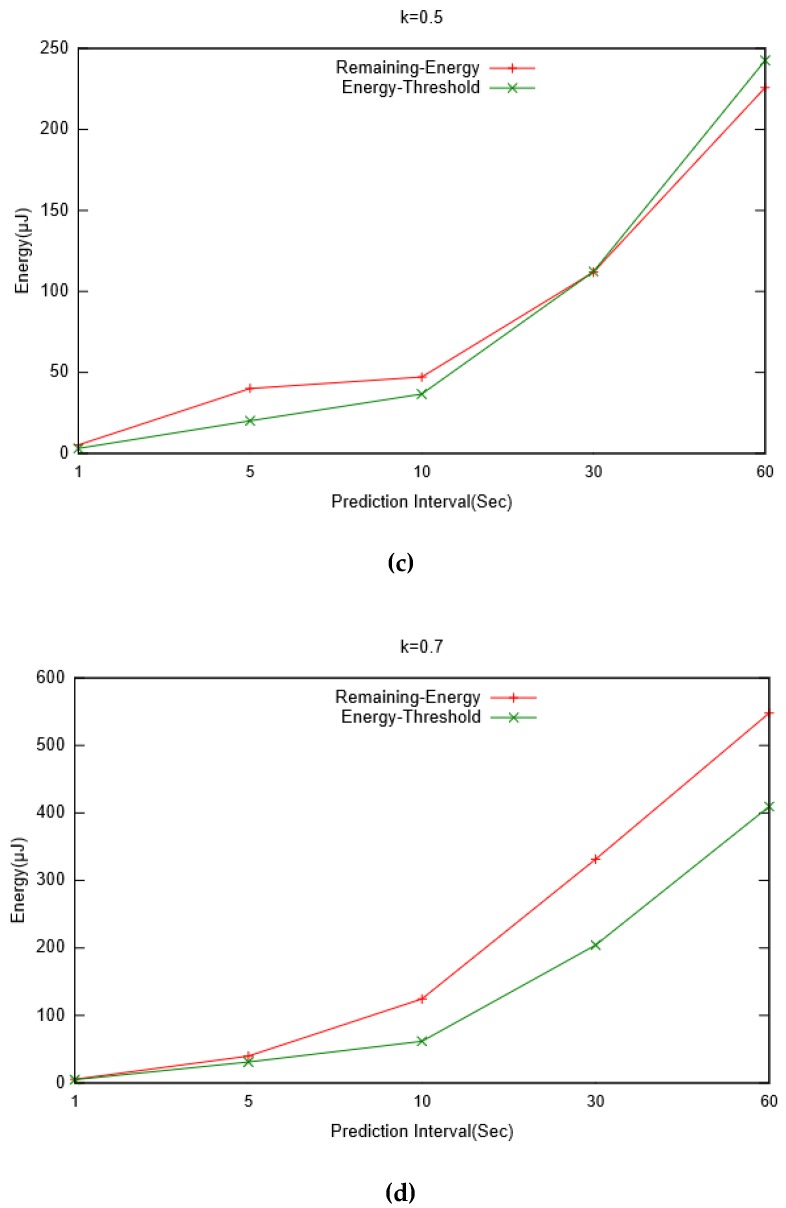
Variations of remaining energy and threshold energy against prediction interval for various values of threshold constant: (**a**) k = 0.1; (**b**) k = 0.3; (**c**) k = 0.5; (**d**) k = 0.7.

The variation of the prediction interval with threshold constant k is presented in Figure 13. It can be observed that for longer prediction intervals, a relatively smaller prediction threshold is required. This clearly points to the trade-off required between a nominal prediction error and the chances that a node runs out of energy before any prediction can be made. Based on this observation, we also compared the remaining energy in buffer (after a prediction is made) with the assumed threshold energy. Figure 14 presents the measurement of remaining energy for different values of prediction intervals observed for various threshold constant k. It can be seen that for greater values of k, remaining energy is well above the threshold limit. On the other hand, for lower values of k, it is either marginal or below the assumed reference. The former is desirable as it would ensure that there is sufficient energy in a node to carry out its operations. However, larger values of k would lead to a smaller prediction interval that would eventually produce greater prediction error.

## 5. Conclusions

In this paper, we present a novel Multi-Node approach for predicting future energy availability for RF-powered WSNs. To the best of our knowledge, no prior work has utilized energy readings from neighbouring nodes in order to make energy predictions. The prediction approach takes into account the harvesting history of all nodes surrounding the main node for which energy prediction is to be made. Once the predicted values corresponding to each node are calculated they are matched with the actual harvested energy and the node giving the least error is taken as the reference node for the next prediction, and so on. In this way, the reference nodes are switched from one node to another for each prediction based on the prediction error. 

The prediction results were compared with SMA (for window size 10) and EWMA (for weighing coefficient 0.4). Different values of prediction interval were considered and MAPE was calculated for each prediction method. The results showed that EWMA followed the energy profile best, while the Multi-Node approach was in between EWMA and SMA. In terms of prediction error, SMA had the lowest MAPE (except for a prediction interval of 1s for which Multi-Node showed the least error) for the considered prediction intervals although it could be observed that the Multi-Node prediction method was less sensitive to prediction intervals than the other two. Overall, it can be said that the Multi-Node prediction approach combines the advantages of both SMA and EWMA, i.e., it follows the energy profile better with less prediction error. Analyses were also made to determine the effective distance up to which a neighbouring node can be considered for prediction, and also the effect of prediction interval on the effective distance. It was found that nodes located at larger distances were utilized less for prediction and as the prediction interval increased the utilization of farther nodes decreased.

We also developed a mathematical model to compute the optimum value of prediction interval, which is essential in designing power management systems for EH-WSNs. The mathematical model was formulated for an RF-EH sensor node with an optimum energy consumption rate considering energy neutrality and minimum energy wastage. A linear relation was established between the prediction interval and the energy threshold (limit before which a prediction should be made). The relation is crucial in determining an optimum prediction interval that would ensure a satisfactory prediction accuracy and a timely prediction before a node runs out of its energy.

## Figures and Tables

**Table 1:** Simulation parameters.

**Source Power**	60 W
**Source Frequency**	2.45 GHz
**Harvester Efficiency**	0.7
**L**	20 m
**d_1_**	1 m
**d_2_**	2.5 m
**d_3_**	5 m
**d_4_**	7 m

**Table 2:** Actual and predicted energy for prediction interval of 60 s.

Time Slots	Actual Harvested Energy (µJ)	Predicted Energy (µJ)			
		SMA	EWMA	Multi-Node (layout-1)	Multi-Node (layout-2)
**15**	2.71	3.07	2.87	3.07	3.07
**25**	2.71	2.91	2.71	2.67	2.78
**35**	3.17	2.55	2.80	2.55	2.55
**45**	3.09	2.75	2.80	2.75	2.77
**55**	2.86	2.91	2.91	2.84	2.78

**Table 3:** MAPE summary table.

Prediction Interval	MAP Error			
	SMA	EWMA	Multi-Node (layout-1)	Multi-Node (layout-2)
**1 s**	1282.32	1375.55	619.60	764.43
**5 s**	48.70	53.66	49.54	51.07
**10 s**	29.10	31.93	32.24	34.05
**30 s**	15.88	17.87	17.83	22.82
**60 s**	8.51	10.42	8.25	10.65

## Abbreviations

AWGN	Additive White Gaussian Noise
EH	Energy Harvesting
ENO	Energy Neutral Operation
EWMA	Exponentially Weighted Moving Average
MA	Moving Average
MAPE	Mean Absolute Percentage Error
RF	Radio Frequency
SMA	Simple Moving Average
WCMA	Weather Conditioned Moving Average
WSN	Wireless Sensor Network
